# Osteotomy at the distal third of tibial tuberosity with LCP L-buttress plate for correction of tibia vara

**DOI:** 10.1186/1749-799X-9-9

**Published:** 2014-02-13

**Authors:** Ye Huang, Jianming Gu, Yixin Zhou, Yujun Li

**Affiliations:** 1 Department of Joint Reconstructive Surgery, Beijing Jishuitan Hospital, Beijing 100035, China

**Keywords:** Tibia vara, High tibial osteotomy, Closing wedge, Locking compression plate, Delayed union

## Abstract

**Background:**

Many osteotomy methods and fixation types have been used to correct the misalignment observed in tibia vara and to achieve a more uniform distribution of weight across the knee joint.

**Purpose:**

The aim of this study is to test the efficacy and safety of a modified closing wedge high tibial osteotomy (CWHTO) procedure for tibia vara.

**Methods:**

In this prospective study, young adults with tibia vara and mild medial arthritic changes were included. A CWHTO was performed at the distal third of the tibial tuberosity, instead of the conventional proximal site. An L-shaped locking compression plate was used for internal fixation. Before/after evaluation of femoro-tibial angle (FTA), pain relief, patellar height, and posterior tibial slope were evaluated. Adverse events were monitored.

**Results:**

Seventy-five knees from 46 patients aged 27.2 ± 5.8 years (range, 14–43 years) underwent the modified CWHTO procedure. After a follow-up of 26.3 ± 7.4 months (range, 15–46 months), FTA correction was 12.4° ± 4.7° (range, 7°–31°), and pain was relieved. Reduction in the posterior tibial slope was 3.0° ± 2.3° (*p* < 0.001), while there was no significant change in patella height. Bone union was observed in all patients. There were a delayed union in four knees, a peroneal nerve lesion in five knees causing partial paralysis and/or sensory loss, and infections in two knees. Three patients underwent a second surgery. All adverse events were successfully treated except for a case of extensor hallucis longus muscle paralysis.

**Conclusions:**

The modified CWHTO procedure is efficient and safe for the correction of tibia vara in young patients.

## Introduction

Osteoarthritis of the knee is a common condition that may occur in young people. In cases of misalignment of the knee joint such as tibia vara, the medial compartment of the knee is submitted to abnormally high forces and weight loading that causes progressive wearing of the joint cartilage and further aggravates the varus deformity. This results in a vicious cycle, which ultimately leads to progressive osteoarthritis if the misalignment is not corrected [1].

Many methods of osteotomy and types of fixation have been used to correct the misalignment and to achieve a more uniform distribution of weight across the knee joint [2,3]. Coventry [4] used a closing wedge osteotomy proximal to the tibial tuberosity, the so-called closing wedge high tibial osteotomy (CWHTO). Recently, new techniques for medial open-wedge high tibial osteotomy (OWHTO) have been popularized. Among these are the locking compression plates (LCP) that are widely used in OWHTO and provide superior initial stability [5-7].

Each method has its pros and cons, and specific considerations when selecting the patients are necessary. The disadvantages of medial OWHTO are that it affects the height of the patella [8-10], is associated with slower bone healing, and requires autograft when the opening width exceeds 13 mm; therefore, simultaneous bilateral osteotomies are not recommended [11]. The disadvantages of CWHTO include adverse effects on patello-femoral (PF) biomechanics, and a limited correction capability [3]. Therefore, for correcting severe deformities, particularly in young patients, there is a need for an osteotomy procedure that does not disturb PF kinematics, allows rapid healing, is possible without the need for a supplementary surgery for harvesting autogenous bone, and can be performed bilaterally simultaneously.

With an aim to meet the above requirements, we tested a modified CWHTO procedure. The modification is that the osteotomy was performed at the distal third of the tibial tuberosity, followed by insertion of an L-shaped LCP plate to facilitate fixation. The distal third of the tibial tuberosity was selected to avoid disturbing the patella tendon and in order to perform the surgery as proximally as possible to obtain a better healing potential in cancellous bone area. The present study aimed to answer two questions: (1) What is the efficacy of this modified approach in terms of correction angle, patella height, tibial slope, Insall-Salvati (IS) index and pain alleviation? (2) What is the safety of this modified approach in terms of consolidation, alignment, re-operation, infections, and neurologic complications? Safety was evaluated from the record of adverse events and complications that are known to occur after conventional CWHTO [12].

## Materials and methods

### Study design

The aim of this prospective study was to assess the efficacy and safety of a modified CWHTO procedure. The surgeries were performed between March 2008 and October 2010 at the Beijing Jishuitan Hospital, Beijing, China. The study was approved by the institutional review board of Beijing Jishuitan Hospital, Beijing, China, and all patients provided a written informed consent. We enrolled tibia vara patients with mild arthritic changes who were admitted for surgical correction of the misalignment. A modified procedure for tibial osteotomy was performed, and an L-shaped LCP plate was inserted for rigid fixation (described below). Patients were followed up for a maximum of 4 years. Standard clinical and radiological methods were used for diagnosis and for assessment of treatment outcomes in terms of limb alignment, pain relief, patella height, tibial slope change, bone healing, and complications.

### Patient selection

The inclusion criteria were as follows: (1) patients younger than 45 years and (2) tibia vara deformity with mild medial-compartmental osteoarthritis. Exclusion criteria were as follows: (1) severe obesity (body mass index (BMI) >40.0 [13]), (2) loss of lateral meniscus, (3) degenerative joint disease in the lateral compartment, (4) limited range of motion of the knee (flexion contracture >10°), or (5) less than two thirds of the articular gap of the medial compartment, as observed by radiography [11,14].

### Preoperative assessments

**Figure 1:**
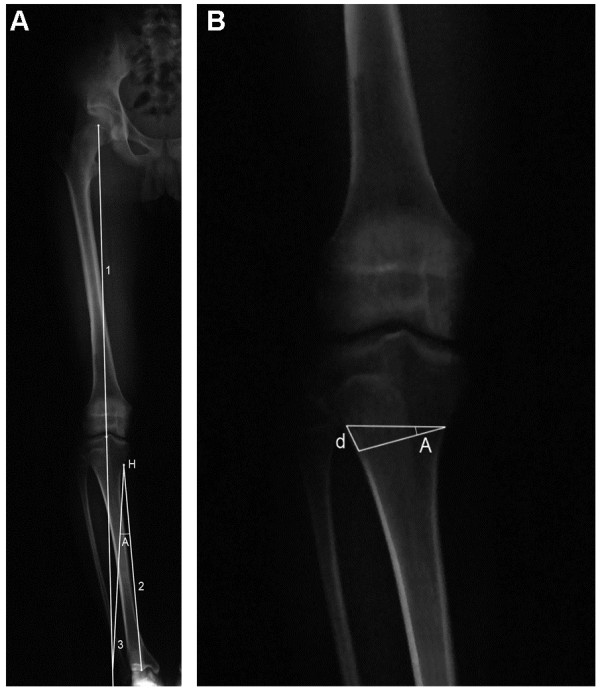
**Miniaci method for frontal plane correction. (A)***Line 1* represents the planned Mikulicz line for the postoperative correction extending from the center of the hip through the center of the knee, past the ankle. *Line 2* connects the osteotomy hinge point (*H*) with the center of the ankle. The *H* point was located on the medial cortex of the proximal tibia at the lower level of tibial tuberosity. With *H* point as the center and the length of *line 2* as the radius, an angular arc is drawn from the center of the ankle to the intersection of *line 1. Line 3* connects the *H* point with the intersection of *line 1*. The angle formed by *lines 2* and *3* is the planned correction angle (*A*). **(B)***Angle A* is now drawn on the prospective site of the osteotomy on the AP radiographs of the knee joint. The distance (*d*) of the two lines at the lateral cortex was measured as the osteotomy wedge base (magnification factor must be considered).

Patients were graded using the Ahlbäck classification [15]. Preoperative planning included measuring the femoro-tibial angle (FTA) on standard anterior-posterior (AP) radiographs of the knee joint and mechanical axis on the standing AP radiographs of the entire lower limbs. The ideal postoperative limb alignment was considered to be 5°–7° of anatomical valgus angulation (FTA, 173°–175°) in order to obtain a mechanical axis (Mikulicz line) of the lower extremity passing 4 ± 2 mm medial to the center of the knee joint [16]. We tried to achieve a Mikulicz line through the center of the knee with minimal overcorrection in young and active adults, instead of obvious valgus overcorrection by the Fujisawa method [14]. The Miniaci method was used to determine the frontal plane correction (Figure 1) [17].

### Surgical procedure

During surgery, the patient was placed in the supine position and a tourniquet was placed high on the thigh. A fibular osteotomy was performed through a 3–4-cm incision made over the lateral aspect of the leg at about 15 cm distal to the fibular head [18]. The length of fibular resection was equal to or shorter than the base of the tibial osteotomy wedge.

**Figure 2:**
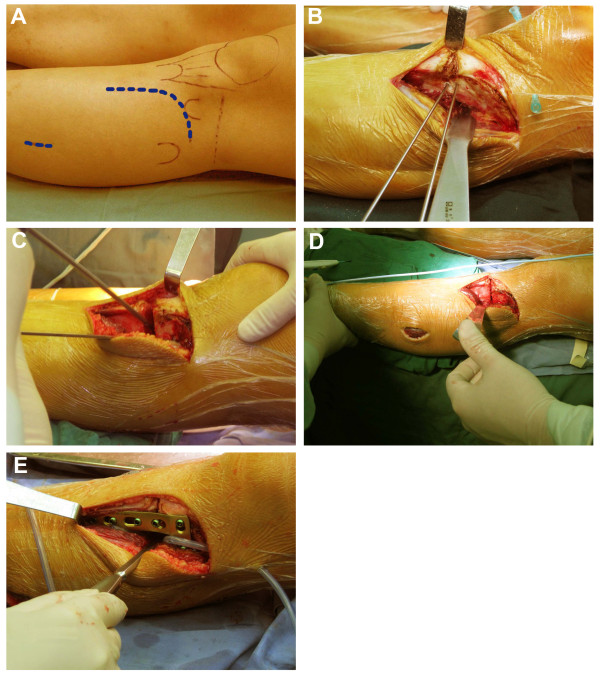
**Surgical procedure for modified CWHTO. (A)** A curvilinear incision was made over the lateral aspect of the proximal tibia, and a 3–4-cm incision was made over the lateral aspect of the leg at the level centered about 15 cm distal to fibular head. **(B)** The extensor muscles were detached to expose the lateral tibial cortex. The osteotomy site was at the distal third of the tibial tuberosity. Two K-wires were drilled in as osteotomy guides. **(C)** The cut was initiated with a saw under constant irrigation and completed with an osteotome. The hinge of the medial cortex was carefully penetrated with the use of a drill instead of an osteotome. **(D)** The wedge was closed and the frontal alignment was checked with a cautery cord. **(E)** An L-shaped LCP plate was inserted for internal fixation. The proximal part of the plate was placed at the level of Gerdy’s tubercle. The shaft of the plate was aligned to the longitudinal axis of the tibia. A full contact between the plate and the bone surface was not necessary.

A curvilinear incision was made over the lateral aspect of the proximal tibia, which extended obliquely from Gerdy’s tubercle to the level of the tibial tuberosity to avoid disturbing the patella tendon, and continued distally 1 cm lateral and parallel to the anterior crest of the tibia (Figure 2A). The extensor muscles were detached to expose the lateral tibial cortex. Two K-wires were drilled in as osteotomy guides. By fluoroscopy, a proximal wire was inserted at the level of the distal third of the tibial tuberosity and parallel to the joint line. The second wire was inserted distally at a distance that was calculated preoperatively, in order to indicate the desired angle of correction, and exited through the medial cortex where the first wire also exited (Figure 2B). The distance between the two wires was confirmed using a sterile ruler, and the angle formed by the wires was checked by fluoroscopy.

The wires served to guide the bone cuts. We began the cuts using a saw under constant irrigation for cooling and then continued with the osteotome. The hinge of the medial cortex was carefully penetrated with the use of a drill instead of an osteotome (Figure 2C). The wedge was extracted completely, and we made sure that no fragment of cortical bone persisted in the posterior part of the osteotomy. The osteotomy was closed slowly and carefully. More than 90% of the hinge could be preserved.

The surgery was performed under fluoroscopic control. The frontal alignment was checked with a cautery cord. One end was placed over the center of the femoral head and the other over the center of the ankle, making sure that the cord was tight and straight. An adequate correction was achieved when the cord passed directly over the center of the knee (Figure 2D). The posterior slope of the tibia was also evaluated under fluoroscopy to prevent the introduction of an anterior slope or excessive slope angle alteration.

If the extracted wedge was too small, the osteotomy surface was cut again. In general, large wedge resection was avoided, but if it was done, the osteotomy gap was only partially closed to avoid overcorrection, and the gap was carefully filled with autograft of a cancellous bone from the resected tibial wedge after the fixation was completed. An L-shaped LCP was used for internal fixation. The plate (Synthes GmbH, Oberdorf, Switzerland) was made of pure titanium, 2.5-mm thickness, 16.0-mm width, and had four or five holes (shaft). The proximal part of the plate was placed at the level of Gerdy’s tubercle or immediately distal to it. The shaft was aligned to the longitudinal axis of the tibia, and the shaft end was attached to the bone surface distally. The plate’s position could be adjusted anteriorly or posteriorly. It was not necessary to bend it to adapt it to the bone surfaces. Three self-tapping 5.0-mm locking head screws were inserted as deep as possible in the proximal segment. In cases where the medial hinge was well-preserved, only two screws were used if the bone ends contacted well. Three more locking screws were inserted distally (Figure 2E).

After fixation, the tension of the intercompartmental septum between the anterior and lateral muscle compartments was assessed by palpation with a finger at the fibula incision. If the septum was tight and the proximal fibula was adducted, we recommend releasing the septum. After using an elevator to elevate the septum of the fibula from distal to proximal about 3–4 cm, we could free the fibula proximal end and place it in the correct position for bone healing. The tourniquet was released, and the wounds were closed. The fascia that covers the anterior tibialis muscle was released with scissors if it was tight after reattaching the anterior tibialis, and drainage was applied.

### Postoperative procedures and follow-up

Patients operated in only one knee started isometric quadricep exercises on the first day and received immediate continuous passive motion (CPM) after drainage removal. Touchdown weight-bearing (15–20 kg) was allowed in the first 6 weeks. After 6 weeks, weight-bearing was increased by 10–15 kg per week. In cases where both knees were operated, weight-bearing was not allowed in the first 6–8 weeks. Patients were then provided with a wheelchair, and deep vein thrombosis (DVT) prophylaxis (compression stockings and warfarin for 4–5 weeks or less) was recommended. Partial weight-bearing was allowed only after 6–8 weeks. In most cases, full weight-bearing was allowed on the second follow-up at the 12th week, when radiographic consolidation had taken place. Follow-up was routinely performed on the 6th, 12th, 24th, and 52nd weeks after operation. Clinical and radiographic assessments were performed at each visit, and knee pain was evaluated using a visual analog scale (VAS). The fixation plate was usually removed 1 to 1.5 years after operation, upon patients’ request.

### Radiographic assessments

Standard AP and lateral radiographs of the knee joint and standing AP radiographs of the entire lower limbs were obtained pre- and postoperatively and were compared. FTA was measured on the AP knee radiograph, and the Mikulicz line was evaluated on the long film of the entire lower limbs. The posterior tibial slope was measured according to the method of Brazier et al. [19] on the true lateral view using the posterior tibial cortex as a reference. The posterior tibial cortex was easily identifiable on radiographs and was used as a reliable landmark for minimizing systematic error [20,21]. IS index was also measured on the lateral radiographs preoperatively and at the last follow-up by measuring the ratio of patellar tendon length to the diagonal length of the patella [22].

### Statistical analysis

SPSS 18.0 (SPSS Inc., Chicago, IL, USA) was used for statistical analysis. FTA, posterior tibial slope, and IS index pre- and postsurgery were compared using paired *t* tests. A *p* value <0.05 was considered statistically significant.

## Results

### Patient characteristics at baseline

The study included 46 patients (17 males and 29 females). Patients were young, with an average age of 27.2 ± 5.8 years (range, 14–43 years) and a BMI of 21.7 ± 3.2 (range, 18.2–30.1) (Table 1). Diagnoses were constitutional tibia vara in 48 knees, idiopathic medial metaphyseal dysplasia in 22 knees, and iatrogenic or traumatic partial closure of the epiphyseal plate in 5 knees (9 left knees, 8 right knees, and 29 bilateral (58 knees)). Medial compartment osteoarthritis changes were mild. In radiological assessments, 13 knees (17.3%) had a narrow medial articular gap, with more than two thirds of residual cartilage being intact. All knees were Ahlbäck grade I. The average preoperative FTA was 186.2° ± 5.1° (range, 179°–209°). The mechanical distal lateral femoral angle was 87.4° ± 2.2° (84°–91°), and the medial proximal tibial angle was 75.8° ± 5.4° (53°–83°). Of the 75 operated knees, 35 (47%) had more than 13° varus deformity.

**Table 1:** Baseline patients’ characteristics

**Patients’ characteristics**	**Values**
Age, years (mean ± SD, range)	27.2 ± 5.8, 14–43
Male/female ratio	17:29
Body mass index (mean ± SD, range)	21.7 ± 3.2, 18.2–30.1
FTA (mean ± SD, range)^a^	186.2° ± 5.1°, 179°–209°
Mechanical distal lateral femoral angle (mean ± SD, range)^a^	87.4° ± 2.2°, 84°–91°
Mechanical medial proximal tibial angle (mean ± SD, range)^a^	75.8° ± 5.4°, 53°–83°
Requiring single knee operation, *n*	17 (17 knees)
Requiring bilateral operation, *n*	29 (58 knees)

^a^Values calculated for 75 knees. *n* = 75 operated knees in *n* = 46 patients.

### What is the efficacy of this modified approach in terms of correction angle, patella height, tibial slope, IS index, and pain alleviation?

**Figure 3:**
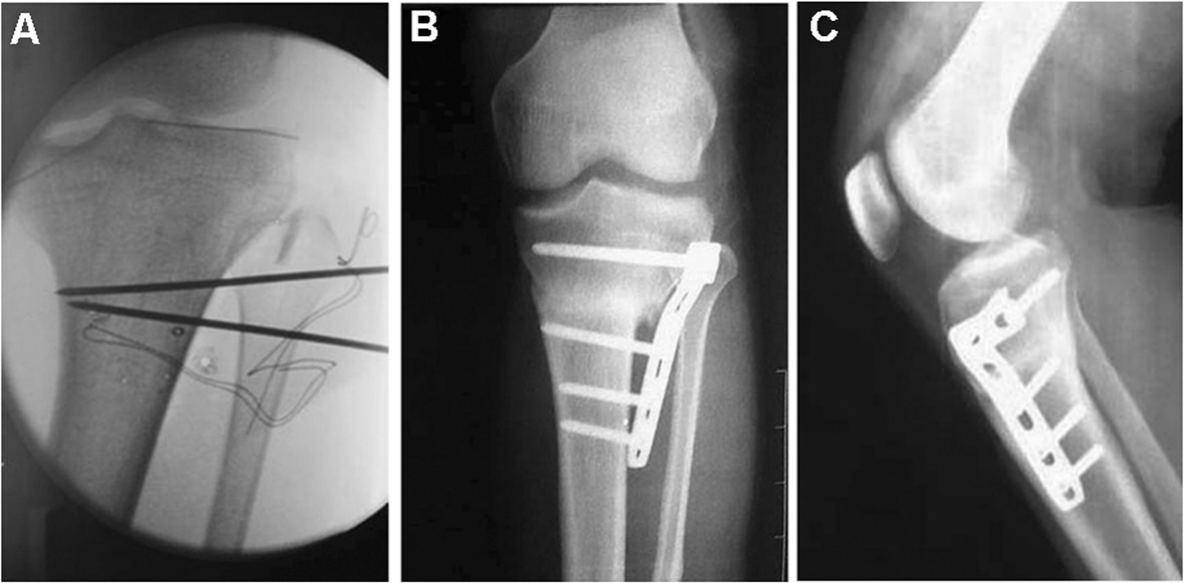
**Modified procedure for CWHTO.** The osteotomy site was at the distal third of the tibial tuberosity. The procedure was performed under fluoroscopy, and an LCP was inserted for internal fixation. **(A)** Two K-wires were intraoperatively drilled in as osteotomy guides. The proximal wire was inserted at the level of the distal third of the tibial tuberosity and parallel to the joint line. The distal wire was then inserted in order to indicate the desired angle of correction and exited through the medial cortex where the first wire exited. The angle was determined from preoperative radiographic assessments and confirmed by fluoroscopy. **(B, C)** AP and lateral radiographs, respectively, 9 months after operation. The proximal fixation screws were at the level of or immediately distal to Gerdy’s tubercle, far from the articular surface. A full contact between the plate and bone surface was not necessary.

**Figure 4:**
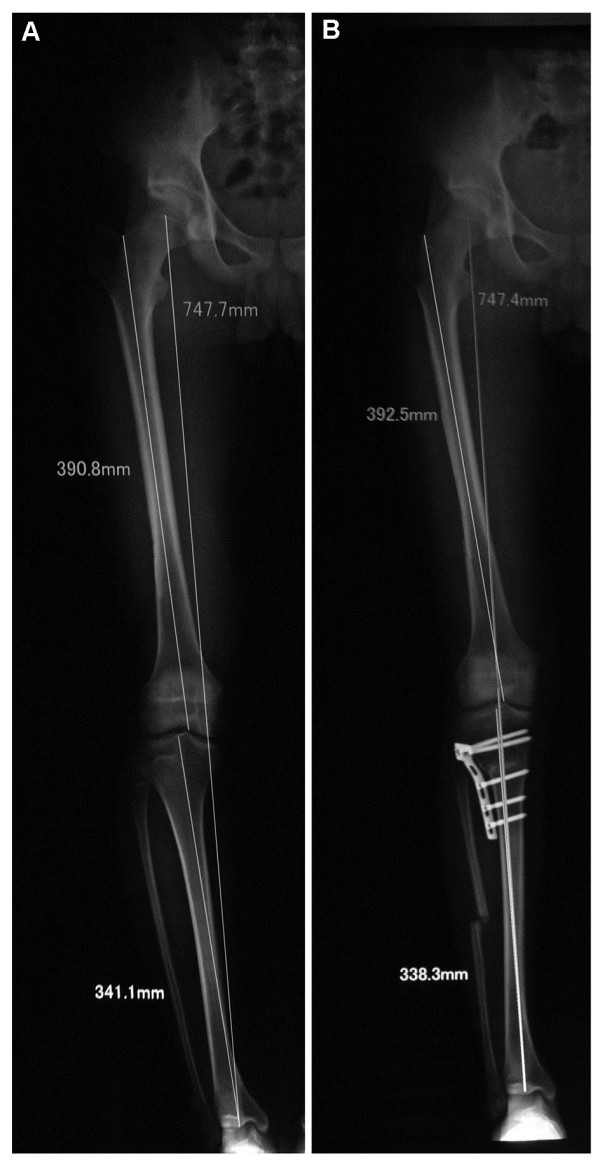
**FTA correction by a modified CWHTO. (A)** Preoperative; **(B)** 12 weeks postoperative. Full-length AP radiographs of a case of osteotomy showing correction of mechanical axis of lower limbs (Mikulicz line).

Postoperative follow-up (mean of 26.3 ± 7.4 months; range, 15–46 months) included clinical and radiological assessments. Five patients were lost to follow-up after plate removal, between the 12th and 18th months after osteotomy. The average postoperative FTA was 173.3° ± 2.0° (range, 170°–178°) (Table 2), and the average correction angle was 12.4° ± 4.7° (range, 7°–31°) (Figures 3 and 4). There was no secondary loss in postoperative alignment in any patient in our study. The mean slope before the operation was 5.9° ± 2.4° (range, 0°–11°) and was corrected to an average of 2.8° ± 1.9° (range, −3° to 7°) after operation. The average reduction in posterior slope was 3.0° ± 2.3°, which was statistically significantly different when comparing before and immediately after operation (*p* < 0.001). The IS index was 1.03 ± 0.13 before and 1.02 ± 0.14 after the operation (*p* = 0.161). Most patients were relieved of their preoperative pain. Average VAS pain score decreased from 2.4 ± 1.6 (range, 1–7) to 0.6 ± 1.0 (range, 0–3).

**Table 2:** Comparison of relevant parameters before and after the modified CWHTO in patients with tibia vara

**Characteristic**	**Before**	**After**	***p *** **value**
Femorotibial angle (range)	186.2° ± 5.1° (179° to 209°)	173.3° ± 2.0° (170° to 178°)	<0.001
Pain^a^	2.35 ± 1.6	0.56 ± 1.0	<0.001
Posterior tibial slope (range)	5.9° ± 2.4° (0° to 11°)	2.8° ± 1.9° (−3° to 7°)	<0.001
Insall-Salvati index (range)	1.03 ± 0.13 (0.8 to 1.3)	1.02 ± 0.14 (0.8 to 1.3)	0.161

Data are presented as means ± SD and were recorded at baseline (before) and at the last follow-up (after) for *n* = 75 operated knees in *n* = 46 patients. Patients were followed up after plate removal for 15–46 months. ^a^Pain was assessed by the VAS pain score.

### What is the safety of this modified approach in terms of consolidation, alignment, re-operation, infections, and neurologic complications?

There was delayed union in four knees in three patients. In three cases, full weight-bearing was not allowed for 6–9 months postsurgery, until radiographic consolidation was observed. One knee was re-operated on the sixth postoperative month, because the patient thought the affected knee was more valgus than desired and that the appearance was asymmetric. In this patient, the alignment was adjusted, the osteotomy site was autografted with cancellous bone from the iliac crest, and the fixation was revised. The bone healed as expected. Infections occurred in two knees (2.6%), which were treated with debridement and external skeletal fixation. The infections were controlled, and the corrected alignment was maintained. Peroneal nerve complications occurred in five knees. Two patients had paralysis of the dorsiflexion muscles innervated by the common peroneal nerve (tibialis anterior, extensor digitorium longus, and extensor hallucis longus in the anterior compartment). Full recovery occurred at 6 months. Sensory loss alone occurred in two knees, both of which recovered within 3–6 months. One patient had extensor hallucis longus muscle paralysis. She did not recover at the latest follow-up (15 months postsurgery).

## Discussion

The patients selected for the present study had several characteristics that prompted us to modify the CWHTO procedure rather than following the conventional CWHTO or OWHTO. Most of the included patients were young and active, requiring avoiding any ill-effects on PF kinematics that are often reported in OWHTO and conventional CWTHO. Thirty-five of 75 knees had more than 13° varus deformity. According to the Hernigou trigonometric chart [23], in OWHTO, the open wedge gap would have been more than 13 mm, which would have required harvesting an autograft from the iliac crest [11]. Twenty-four of 29 patients with bilateral deformities expected simultaneous bilateral operation. This is not recommended in OWHTO because of the risk of secondary loss of correction caused by overloading on the bones that consolidate slowly [11]. Hence, we modified the conventional CWTHO procedure to meet the requirements of this group of patients and tested its efficacy and safety.

### What is the efficacy of this modified approach in terms of correction angle, patella height, tibial slope, IS index, and pain alleviation?

In this study, the correction was accurate, as seen from the postoperative FTA (mean, 173.3° ± 2.0°, range, 170°–178°) (Table 2). In elderly patients with progressive osteoarthritis, the alignment usually requires an overcorrection, such that the weight-bearing axis passes through a point located lateral on the tibia plateau by the Fujisawa scale [14]. Such a degree of valgus alignment is unacceptable in young patients who expect a normal appearance and full activity. Hence, we tried to achieve minimal overcorrection with the Mikulicz line passing through the center of the knee. Most patients have been relieved of their preoperative pain. The long-term results of the affected knees need to be followed up and studied in the future.

Stoffel et al. [24] compared medial open wedge osteotomies that were either distal or proximal to the tuberosity and found that PF contact stress was significantly lower in the distal osteotomy group. The osteotomy site in the modified CWHTO procedure was distal to the tuberosity. The difference in the IS index before (1.03 ± 0.13) and after (1.02 ± 0.14) the operation was not statistically significant. Thus, this procedure did not change the patellar height. In the present study, all cases had minimal cartilage loss in the medial compartment. The rigid internal fixation and early mobilization of the knee used in this procedure also contributed to the preservation of normal PF kinematics.

Both open and closing wedge osteotomies primarily involve the frontal plane but also affect the posterior tibial slope. CWHTO is known to reduce the tibial slope by approximately 5° [25]. In the present study, the average reduction in posterior slope was 3.0° ± 2.3°. However, if the tibial slope changes less than 5°, it does not significantly alter the *in situ* forces on the cruciate ligament [26,27]. The smaller effect (average 3°) on posterior tibial slope might be because it is different from the triangle geometry at the conventional osteotomy site [28], as the lateral cortex of tibia is more parallel to the sagittal plane at the level of the distal third of tuberosity. When the wedge is resected, the anterior and posterior closures on the sagittal plane are almost equal, and the slope changes little. Fluoroscopic examination also helped to assess the slope change after wedge closing. It is important to note that the amount of bone resected was equal anteriorly and posteriorly, making it prone to anterior sloping. Therefore, a careful X-ray monitoring should be performed.

### What is the safety of this modified approach in terms of consolidation, alignment, re-operation, infections, and neurologic complications?

Compared with a conventional CWHTO or OWHTO, the osteotomy site was more distal in the present study. The usual concern when an osteotomy is made at this level is a risk of a delayed bone union or non-union [29], which was not the case in the present study. Indeed, radiographic consolidation was observed within 3 months in most patients in our study. There are several reasons to explain this high union rate. Because all included patients were young (average age about 27 years), they presumably had a greater healing potential, and the soft tissues surrounding the osteotomy site were strong. The osteotomy site was as proximal as possible, although not above, the tibial tuberosity. At this site, two broad cancellous surfaces retain an excellent healing potential when they are in a stable, direct apposition. The technical factors that are likely to have contributed to the high union rate include avoidance of complete fracture of the medial cortex and retaining the medial soft tissue hinge intact. With the stable medial hinge, the locking plate can provide stable initial fixation. We also autografted the osteotomy site with cancellous bone from the resected tibial wedge if there was a gap.

Many studies have reported good outcomes of using an LCP in OWHTO [30]. The use of an LCP in CWHTO also provided reliable fixation in the present study. There was no secondary correction loss in postoperative alignment in any patient. The LCP also simplified the fixation process. It is unnecessary to bend the plate for implant contouring, because the principle of the locking plate does not require form-fit seating on the bone. The proximal screws at the level of Gerdy’s tubercle allowed the soft tissues close to the joint capsule to be kept intact, including the attachment of the iliotibial band. The screws were distant enough from the joint to prevent articular penetration.

Peroneal nerve lesion occurred in 5 out of 75 operated knees, due to the fibula osteotomy that is part of this modified CWHTO procedure. In our experience, patients with more severe tibia vara deformity, especially those who have greater tibia torsion, are at higher risk of peroneal nerve palsy. The nerve may be injured by entrapment in the intercompartmental septum between the anterior and lateral muscle compartments or due to high tension in the compartmental fascia. We selected the site of the fibular osteotomy in the middle portion of the fibular shaft (15 cm distal to the fibula head [18]) to prevent injury of the common peroneal nerve and its branches. In cases of serious deformity, the intercompartmental septum could be twisted after correction, because our osteotomies on tibia and fibula were not on a same level. The high tension caused by the swelling of the muscles and the hematoma in the anterior compartment could not be released, which probably caused nerve injury or a kind of anterior compartmental syndrome. Therefore, we strongly recommend verifying the tension in the intercompartmental septum by touch. If the septum is tight and the proximal fibula is stuck in an adducted position, we can elevate the septum and free the proximal end of the fibular bone. In addition, we also recommend releasing the fascia that covers the anterior tibialis muscle with scissors if it is tight after suturing the attachment of the anterior tibialis.

## Conclusion

In conclusion, the modified CWHTO procedure described in the present study, in which the osteotomy was performed at the level of the distal third of the tibia tuberosity, was found to be safe and effective in correcting the varus deformity, relieving pain and preserving PF kinematics. Compared with OWHTO, complications of a fibula resection is still a concern. This procedure may be used for young and active patients with low to severe tibia vara, both uni- or bilateral, and low to mild OA. In future studies, a longer follow-up may be included to study the long-term activity and function of the affected joints.

## Competing interests

The authors declare that they have no competing interests.

## Authors’ contributions

YH carried out the study, participated in the collection of data, and drafted the manuscript. JG performed the statistical analysis and participated in its design. YZ and YL helped draft the manuscript. All authors read and approved the final manuscript.
